# Bridging Inflammation and Repair: The Promise of MFG-E8 in Ischemic Stroke Therapy

**DOI:** 10.3390/ijms26178708

**Published:** 2025-09-06

**Authors:** Ye-Jin Han, Hye-Jin Lee, Dong-Ho Geum, Jong-Hoon Kim, Dong-Hyuk Park

**Affiliations:** 1 Laboratory of Neurosurgery, Department of Biomedical Sciences, College of Medicine, Korea University, Seoul 02841, Republic of Korea; yjhan1224@korea.ac.kr (Y.-J.H.); hjion2344@korea.ac.kr (H.-J.L.); 2 Department of Neurosurgery, Anam Hospital, College of Medicine, Korea University, Seoul 02841, Republic of Korea; 3 Department of Convergence Medicine, College of Medicine, Korea University, Seoul 02841, Republic of Korea; geumd@korea.ac.kr; 4 Laboratory of Stem Cells and Tissue Regeneration, Department of Biotechnology, College of Life Sciences and Biotechnology, Korea University, Seoul 02841, Republic of Korea; jhkim@korea.ac.kr

**Keywords:** ischemic stroke, lactadherin, MFG-E8, neuroinflammation, efferocytosis

## Abstract

Ischemic stroke is a neurological disorder resulting from localized brain injury due to focal cerebral ischemia, typically caused by the blockage of one or, in some cases, a few cerebral arteries. This arterial obstruction leads to hypoxia and energy failure, culminating in primary brain damage. Although reperfusion is critical to salvage viable tissue, it often intensifies injury through oxidative stress, inflammation, and cell death—a phenomenon called ischemia–reperfusion (I/R) injury. Milk fat globule-EGF factor 8 (MFG-E8), a multifunctional glycoprotein secreted by stem and immune cells, is a key regulator of inflammation and tissue repair. By modulating microglial activation, attenuating proinflammatory cytokine releases, and preserving neuronal integrity, MFG-E8 mitigates ischemia–reperfusion injury and emerges as a novel therapeutic target for ischemic stroke.

## 1. Introduction

Ischemic stroke is the second leading cause of death and a major cause of disability worldwide, causing significant social and economic burdens [1,2,3,4]. It accounts for approximately 87% [5] of all stroke cases and is predominantly caused by such focal arterial obstructions [6]. Extensive cellular necrosis occurs in the ischemic core, while in the surrounding penumbra region [7]. Complex secondary damage ensues, including the activation of reactive glial cells and the breakdown of the blood–brain barrier (BBB) [8]. Current standard treatments include intravenous thrombolysis with recombinant tissue plasminogen activator (r-tPA), which must be administered within a narrow 4.5 h therapeutic window after symptom onset, and mechanical thrombectomy, which can be performed in selected patients up to 24 h after onset. Despite these advances, the restricted therapeutic windows still limit the proportion of patients who can benefit from these interventions [9,10,11,12,13,14]. Furthermore, despite advancements in medical infrastructure and administration of treatment within the recommended time frame [15], clinical improvement often falls short of expectations, with low reperfusion success rates of approximately 35% [16,17].

Serious side effects, such as intracranial hemorrhage, reperfusion injury, and long-term neurological deficits, can occur [18,19,20]. The severe limitations of current ischemic stroke interventions underscore the urgent need for novel therapeutic strategies with longer treatment windows and neuroprotective effects that ultimately improve prognosis [21,22,23,24]. Milk fat globule-EGF factor 8 (MFG-E8) has emerged as a promising biomolecule in stroke research [25,26,27]. MFG-E8, also known as lactadherin, is a multifunctional glycoprotein originally identified in milk fat globules and is constitutively expressed in various tissues, including the brain, liver, kidneys, and immune cells [28]. Initially recognized as a “bridging” protein mediating the phagocytic clearance of apoptotic cells [29,30,31,32], MFG-E8 has multifaceted physiological functions, including inflammation suppression, tissue homeostasis maintenance, and tissue repair promotion [33,34]. Specifically, in ischemic brain injury models, MFG-E8 expression has been reported to increase primarily in the ischemic penumbra rather than in the infarct core [25]. Cheyuo et al. [27] demonstrated that intravenous administration of exogenous recombinant human MFG-E8 (rhMFG-E8) one hour after ischemia resulted in reduced infarct volume and improved neurological function at 24 and 48 h post-ischemia. Histological analysis confirmed that rhMFG-E8 protected neurons from necrosis in the ischemic penumbra. Additionally, Choi et al. [25] reported that animals treated with MFG-E8 showed a significant decrease in Iba-1-positive cells and an increase in RECA-1-positive cells in the peri-infarct region. Furthermore, the number of 5-Bromo-2′-deoxyuridine (BrdU)- and Doublecortin (DCX)-positive cells in the subventricular zone was significantly higher in the MFG-E8-treated group compared to controls. These findings suggest that MFG-E8 reduces inflammation, promotes angiogenesis, and enhances neurogenesis in ischemic models.

It exerts neuroprotective effects through various pathophysiological mechanisms, including enhanced phagocytosis by macrophages and microglia, modulation of reactive astrocytes [35,36,37], upregulation of angiogenesis [38], and enhanced neuroregeneration [25,26,27,28]. These multifaceted mechanisms demonstrate the potential of MFG-E8 as a next-generation multifunctional therapeutic that can target multiple pathways simultaneously.

Although several original research articles have investigated MFG-E8 in the context of ischemic stroke, most existing review articles have either focused on its general biological functions or explored other pathological conditions. Comprehensive reviews that emphasize ischemic stroke specifically, while integrating molecular structural biology with mechanistic insights into stroke pathology, remain limited. This review aims to fill this gap by providing an in-depth analysis of MFG-E8’s structural characteristics, its diverse mechanistic roles in ischemic brain injury, and the therapeutic potential that arises from these insights. By thoroughly examining the inflammatory responses and secondary damage mechanisms underlying ischemic stroke pathology, we propose new therapeutic strategies targeting MFG-E8 that may overcome current treatment limitations and ultimately improve patient outcomes.

## 2. Pathophysiological Mechanisms and Signaling Pathways of Ischemic Stroke

Ischemic stroke (IS) arises from diverse mechanisms, including cardioembolism, focal arterial thrombosis in an atherosclerotic plaque, and artery-to-artery embolism originating from such thrombi. Additionally, small artery disease, arterial dissection, vasculitis, prothrombotic states, and hemodynamic disturbances contribute to IS pathogenesis. These events cause localized infarctions in the brain, spinal cord, or retina, leading to neurological deficits [39]. The most common clinical manifestations observed in approximately 80% of patients are motor impairments involving the face, arm, and leg [40,41]. Current therapeutic options include intravenous administration of r-tPA within 4.5 h of symptom onset [42,43,44] and mechanical thrombectomy (MT) for large vessel occlusions [45,46]. However, only a limited number of patients receive early intervention, partly due to restricted access and narrow therapeutic windows [10,11,12,13,14].

**Figure 1:**
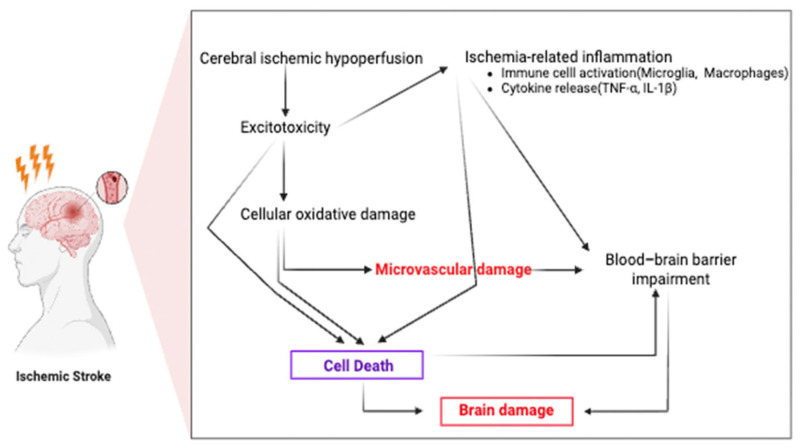
Pathophysiological mechanisms of ischemic stroke. Cerebral ischemia induces hypoperfusion, excitotoxicity, oxidative damage, microvascular dysfunction, and blood–brain barrier breakdown, which contribute to brain injury and inflammation.

Ischemic brain injury progresses through a temporally defined sequence of overlapping phases, spanning from hyperacute to chronic stages [47]. During the hyperacute phase (≤12 h), excitotoxicity and oxidative stress rapidly develop and peak, driving early neuronal damage. The acute phase (12–24 h) follows, characterized by progressively intensifying inflammatory cascades and microvascular dysfunction, which exacerbate tissue injury. In the subacute phase (2 days–2 weeks), injured cerebral tissue releases cytokines, chemokines, adhesion molecules, and matrix metalloproteases (MMPs), leading to increased blood–brain barrier (BBB) permeability and recruitment of peripheral leukocytes, thereby amplifying the inflammatory response. Apoptotic cell death also becomes increasingly prominent, while reparative processes such as angiogenesis and neuroplasticity gradually emerge [48]. Finally, the chronic phase (2 weeks–2 months) involves resolution of inflammation and initiation of tissue repair, with a gradual re-establishment of homeostasis and suppression of inflammatory signaling. However, endogenous repair mechanisms alone are often insufficient to achieve full functional recovery in stroke patients [49,50,51]. The temporal ordering and partial overlap of these injury and repair processes are summarized in Figure 1 [47,48,49,50,51].

Despite advances in reperfusion therapies, including intravenous thrombolysis and MT, clinical application is limited by narrow therapeutic windows and strict eligibility criteria, leaving many patients without effective treatment options. Furthermore, even after successful reperfusion, I/R injury can exacerbate infarct size and worsen clinical outcomes [52]. Without timely and appropriate intervention, the ischemic cascade culminates in irreversible neuronal death and structural brain damage, often resulting in permanent disability [47,48,49,50,51,52,53,54].

These challenges underscore the urgent need for a deeper understanding of the sequential pathophysiological mechanisms underlying ischemic injury, which is essential for developing more effective therapeutic strategies and improving long-term patient outcomes. In this context, MFG-E8 emerges as a key regulator, linking critical injury-related processes such as efferocytosis and microglial polarization. Recent evidence suggests that MFG-E8 can modulate these core pathophysiological pathways, thereby influencing the progression of ischemic injury. These mechanistic insights provide a conceptual bridge between the pathophysiological framework described in this section and the mechanistic and therapeutic perspectives elaborated in Section 3, Section 4 and Section 5, highlighting MFG-E8 as a potential therapeutic target.

### 2.1. Signaling Pathways in Ischemic Stroke: Excitotoxicity

Ischemic stroke is initiated by a sudden reduction in cerebral blood flow, causing oxygen and glucose deprivation, and, ultimately, neuronal energy depletion and impaired Adenosine Triphosphate (ATP) production [55]. Sodium–potassium adenosine triphosphatase (Na^+^/K^+^-ATPase) dysfunction disrupts ionic gradients and induces neuronal depolarization. Consequently, ischemic neurons and surrounding cells release an excessive amount of glutamate into the synaptic cleft, leading to its accumulation in the extracellular space [52,56]. This elevated extracellular glutamate overstimulates ionotropic glutamate receptors on postsynaptic neurons, including N-methyl-D-aspartate receptors (NMDARs), α-amino-3-hydroxy-5-methyl-4-isoxazolepropionic acid (AMPA) receptors, and kainate receptors [53,57,58]. Overactivation of NMDARs causes a pathological influx of calcium ions (Ca^2+^) into the postsynaptic neurons. This excessive intracellular calcium overload triggers excitotoxicity characterized by mitochondrial dysfunction, reactive oxygen species (ROS) generation, activation of calpains, and initiation of downstream cell death pathways [57,59].

NMDARs exhibit dual functions in neuronal survival and death, depending on their subunit composition and subcellular localization. Synaptic NMDARs containing the *GluN2A* subunit promote neuronal survival by mediating Ca^2+^ influx and activating prosurvival signaling pathways, such as Phosphoinositide 3-Kinase (PI3K)/Protein Kinase B (Akt), Extracellular signal-regulated kinases (ERK), and cAMP response element-binding protein (CREB) [59,60,61]. Subsequently, these pathways induce the expression of anti-apoptotic genes, including brain-derived neurotrophic factor (BDNF) and B-cell lymphoma 2 (*Bcl-2*) [62]. In contrast, extrasynaptic NMDAR predominantly composed of *GluN2B* subunits are associated with neurotoxicity. Their activation induces CREB deactivation, proapoptotic gene upregulation, and mitochondrial dysfunction, thereby facilitating neuronal cell death [63,64].

Ischemic conditions promote pathological changes, such as increased extracellular potassium (K^+^) concentrations, reduced pH, and widespread ionic imbalance, further enhancing glutamate release. Furthermore, impaired intercellular communication—such as dysregulated gap junction activity—facilitates the propagation of neuronal injury across adjacent cells and tissues [65].

The prosurvival signaling cascade mediated by synaptic NMDARs is critical in neuronal protection [57]. The PI3K/Akt pathway is activated using calcium/calmodulin, 3-Phosphoinositide-dependent protein kinase-1 (PDK1), and Insulin receptor substrate-1 (*IRS-1*), leading to Akt phosphorylation and activation [66,67]. Activated Akt subsequently inhibits key proapoptotic factors, including glycogen synthase kinase 3 (GSK3β), apoptosis signal-regulating kinase 1 (*ASK1*), and *p53*, thereby preventing cell death [47,68,69,70,71]. Concurrently, CREB phosphorylation using the Ras-ERK-CREB pathway induces BDNF expression, which supports neuronal function and enhances resistance to ischemic injury [72,73,74,75,76,77]. Ischemia experimental models have demonstrated that these signaling pathways provide robust neuroprotective mechanisms against excitotoxic damage [78,79].

In contrast, extrasynaptic NMDARs serve as central mediators of pathological signaling. GluN2B-containing NMDARs are associated with pro-death pathways involving phosphate and tensin homolog (*PTEN*), death-associated protein kinase 1 (*DAPK1*), and the Postsynaptic density protein 95 (PSD95)/neuronal nitric oxide synthase (nNOS) complex. These pathways inhibit the PI3K/Akt survival cascade while promoting nitric oxide (NO) production, oxidative stress, and DNA damage, thereby facilitating neuronal cell death. Notably, DAPK1 interferes with prosurvival signaling by sequestering ERK in the cytoplasm, whereas the PSD95/nNOS complex intensifies injury by enhancing calcium influx and NO generation [80,81,82,83,84,85].

Therapeutic strategies targeting GluN2B-containing or specifically extrasynaptic NMDARs have been proposed to counteract these deleterious processes [86]. One such agent is memantine, a low-affinity, noncompetitive NMDAR antagonist that preferentially inhibits extrasynaptic receptors without significantly affecting physiological synaptic transmission [87]. However, the binary distinction between synaptic and extrasynaptic NMDARs in terms of function and localization is an oversimplification. Therefore, future therapeutic approaches should precisely target the structural and spatial characteristics of NMDARs to achieve selective neuroprotection.

### 2.2. Signaling Pathways in Ischemic Stroke: Mitochondrial Dysfunction

Mitochondria are intracytoplasmic double-membraned organelles crucial for ATP production and calcium signaling, regulating their shape and function through fusion and fission. By generating ATP via glucose- and oxygen-dependent oxidative phosphorylation, they serve as metabolic hubs essential for cell survival and function. During ischemic stroke, mitochondrial dysfunction promotes cell death, making mitochondria key targets for neuronal survival and neuroprotective therapy [88]. However, under pathological conditions such as ischemic stroke, where energy supply is abruptly interrupted, mitochondrial function is severely compromised, causing various forms of cellular stress [89]. Inhibiting ATP production diminishes the mitochondrial membrane potential and induces excessive accumulation of intracellular Ca^2+^, which in turn induces the opening of the mitochondrial permeability transition pore [90,91,92]. This facilitates the release of cytochrome c into the cytosol, activating the intrinsic apoptotic pathways [93,94].

Furthermore, the ischemic environment promotes pathological ROS accumulation, intensifying oxidative stress [95]. This effect is further intensified during reperfusion, contributing to persistent cytotoxicity and secondary tissue damage. Thus, mitochondrial dysfunction is a critical nexus in the pathophysiological cascade of ischemic stroke that links energy failure to cell death mechanisms and long-term neurological impairment [96].

Mitochondrial dysfunction interacts with various intracellular signaling pathways to modulate ischemic brain injury progression [97]. One key pathway involves hypoxia-inducible factor-1 alpha (*HIF-1α*), which facilitates metabolic adaptation by upregulating glycolytic genes under hypoxic conditions [98,99]. However, elevated ROS levels stabilize HIF-1α, creating a positive feedback loop intensifying oxidative stress [100].

In contrast, nuclear factor erythroid 2-related factor 2 (*Nrf2*) exerts a protective role by inducing the expression of antioxidant response element (ARE)-driven genes, thereby promoting ROS detoxification and enhancing cellular defense mechanisms [101]. Nrf2 activation is further supported by upstream signaling cascades, such as PI3K/Akt and ERK/mitogen-activated protein kinase (MAPK), which amplify its antioxidant and cytoprotective effects. The dynamic interplay among these pathways critically influences neuronal survival and the extent of ischemia-induced damage [102].

Casein kinase 2 (CK2) performs a dual role in the context of ischemic injury. It promotes antioxidant defense and angiogenic responses by modulating downstream targets, such as Rac Family Small GTPase 1 (*Rac1*), Signal transducer and activator of transcription 3 (STAT3), and Nuclear factor kappa B (NF-κB). Paradoxically, under overactivation conditions, CK2 facilitates cell death by promoting the release of apoptosis-inducing factors from mitochondria, highlighting its context-dependent functionality [102,103,104].

Meanwhile, mitophagy, which is specifically mediated by *PTEN*-induced kinase 1/Parkin and *Bcl2*-interacting protein 3, is a crucial process in maintaining cellular homeostasis that selectively removes damaged mitochondria [105]. This process prevents excessive ROS accumulation and mitigates mitochondrial-driven toxicity. Mitophagy is especially important during I/R injury, where the efficient clearance of dysfunctional mitochondria can significantly reduce secondary damage and promote neuronal survival [106].

In conclusion, mitochondrial dysfunction and its intricate interplay with multiple signaling pathways orchestrate the pathophysiology of ischemic stroke. These pathways constitute a critical molecular axis that governs the balance between neuronal survival and death. Elucidating and modulating these pathological mechanisms is essential for the development of effective therapeutic strategies. Future research should explore integrated, signaling-targeted approaches to preserving mitochondrial function and mitigating oxidative stress. Such strategies would significantly enhance neuronal survival and facilitate long-term recovery following cerebral ischemia.

### 2.3. Signaling Pathways in Ischemic Stroke: Autophagy

Autophagy is the process by which cells engulf cytoplasmic proteins and damaged organelles, delivering them to lysosomes for degradation. It serves as a key mechanism for removing old components and provides nutrients and energy during stress conditions [107]. Autophagy is an evolutionary conserved self-protective mechanism that is crucial to maintaining cellular homeostasis. It facilitates the degradation of long-lived proteins, misfolded protein aggregates, and damaged organelles, thereby regulating energy balance and enabling cellular adaptation to stress. Recent studies have investigated the activation of autophagy in various cell types, such as neurons, astrocytes, and endothelial cells, following ischemic stroke [107,108].

Moderate levels of autophagy promote neuronal survival by removing toxic protein aggregates and damaged cellular components. However, excessive or dysregulated autophagy facilitates a pathological process that promotes cell death. This dual nature underscores the context-dependent role of autophagy in ischemic injury [109].

Mechanistic target of rapamycin (mTOR) is a central regulator of autophagy that senses intracellular nutrient availability and stress levels, primarily suppressing the initiation of autophagy under normal or nutrient-rich conditions. In ischemic injury, two major pathways, PI3K/Akt/mTOR and AMP-activated protein kinase (AMPK)/mTOR, exert opposing influences on autophagy; the former inhibits autophagy, while the latter promotes it [102]. AMPK is activated in response to energy depletion, leading to the inhibition of mechanistic/mammalian target of rapamycin complex 1 (mTORC1) and stimulation of autophagy through calcium-dependent calmodulin signaling [48,110,111].

The MAPK/ERK pathway also demonstrates context-dependent effects, specifically, either suppressing or promoting autophagy by activating or inhibiting mTOR, respectively, using ERK-mediated mechanisms [102]. In parallel, Beclin1 is crucial in the formation of autophagosomes, with its interaction with the anti-apoptotic protein Bcl-2 as a key regulatory node. In ischemic stroke, Bcl-2 phosphorylation or peroxisome proliferator-activated receptor gamma (PPAR-γ) activation can disrupt or modulate the Bcl-2–Beclin1 interaction, thereby influencing autophagy induction and progression [112,113].

### 2.4. Signaling Pathways in Ischemic Stroke: Cell Death

Ischemic stroke, caused by a disruption of blood flow to the brain, can lead to cell death through various programmed cell death pathways. These pathways include apoptosis, necroptosis, and pyroptosis, all of which contribute to the progression of ischemic damage. In ischemic stroke, energy depletion and disruption of oxygen and nutrient supply lead to extensive neuronal death, primarily through programmed cell death known as apoptosis. Apoptosis is a regulated, genetically controlled process distinct from necrotic cell death caused by cytotoxic injury. It proceeds via two main pathways: the extrinsic (death receptor-mediated) and intrinsic (mitochondria-mediated) pathways [114,115].

When tumor necrosis factor alpha (TNFα) binds to tumor necrosis factor receptor 1 (TNF-R1), TNF-R1 forms a trimer and recruits complex I, which consists of TNF receptor-associated death domain (TRADD), cellular inhibitor of apoptosis proteins (cIAPs), receptor interacting protein 1 (RIP1), and TNF receptor-associated factors 2 and 5 (TRAF2/5). Complex I then forms complex II by recruiting Fas-associated protein with a death domain (FADD), receptor interacting protein 3 (RIP3), and procaspase-8. Within complex II, procaspase-8 (pro-Casp8) is cleaved to active caspase-8 (Casp8). Casp8 cleaves BID into truncated BID (tBID), which activates Bcl-2-associated X protein (*Bax*) to form pores in the mitochondrial outer membrane, inducing the release of cytochrome c (CYT C) and promoting apoptosis [116].

In contrast, the intrinsic pathway is triggered by intracellular stress signals, such as calcium overload and oxidative stress, which cause mitochondrial outer membrane permeabilization [114]. This induces the release of proapoptotic factors, such as cytochrome c, into the cytosol, where they bind to Apoptotic protease activating factor 1 (*Apaf-1)* and procaspase-9 (pro-Casp9) to form the apoptosome, thereby activating caspase-9 (Casp9) and downstream effector caspases (e.g., caspase-3) [117,118]. Together, these cascades amplify neuronal death in the ischemic brain and significantly contribute to infarct progression and neurological dysfunction.

The tumor suppressor protein *p53* plays a pivotal role in neuronal apoptosis in ischemic stroke. Activated by DNA damage, hypoxia, and oxidative stress, p53 modulates the transcription of numerous proapoptotic genes, including *Bax*, in the ischemic region, thereby promoting mitochondrial-dependent apoptosis [119,120,121]. Furthermore, p53 interacts with multiple signaling cascades, including the Notch pathway, where crosstalk among Notch1, p53, NF-κB, and HIF-1α forms a complex regulatory network contributing to neuronal death [122,123]. Inhibition of Notch signaling by γ-secretase inhibitors attenuates microglial activation and confers neuroprotection, highlighting its therapeutic relevance in stroke [123].

Beyond classical apoptosis, nonapoptotic forms of cell death contribute significantly to ischemic brain injury [124]. Pyroptosis is a form of proinflammatory programmed cell death driven by inflammasome formation and caspase-1 activation, leading to the release of inflammatory cytokines, such as Interleukin-1 beta (IL-1β) and Interleukin-18 (IL-18) [125]. Meanwhile, ferroptosis, a unique form of regulated cell death driven by iron-dependent lipid peroxidation, is triggered by intracellular iron overload, glutathione depletion, and impaired glutathione peroxidase 4 (GPX4) activity [126]. Notably, BBB disruption in stroke enhances neuronal iron influx, thereby accelerating ferroptosis. Calcium signaling and the Kelch-like ECH-associated protein 1 (*Keap1*)–*Nrf2* antioxidant pathway regulate this process, which further implicates redox imbalance in cell death [127].

Collectively, these diverse cell death mechanisms reflect the multifaceted nature of ischemic brain injury and underscore the potential of therapeutic interventions targeting pathways such as p53, Notch, pyroptosis, and ferroptosis.

### 2.5. Signaling Pathways in Ischemic Stroke: Neuroinflammation and Microglia

Tumor necrosis factor alpha (TNF-α) is an extensively studied proinflammatory cytokine in ischemic stroke and a critical mediator of neuroinflammatory responses. TNF-α exists in two biologically active forms: soluble TNF (solTNF) and transmembrane TNF (tmTNF), which primarily signal through TNF receptor 1 (TNFR1) and TNF receptor 2 (TNFR2), respectively [128]. The solTNF–TNFR1 axis is associated with proapoptotic and proinflammatory signaling, thereby exacerbating neuronal injury and BBB breakdown. In contrast, the tmTNF–TNFR2 pathway promotes cell survival, neuroregeneration, and anti-inflammatory responses, indicating functional divergence between the two receptors [129].

Microglia are the principal source of TNF-α in the ischemic brain. Experimental studies have shown that inhibiting solTNF signaling can mitigate ischemic brain damage, whereas promoting tmTNF signaling may confer neuroprotective effects. This dichotomy underscores the importance of the selective targeting of TNF-α pathways for therapeutic purposes [130].

**Figure 2:**
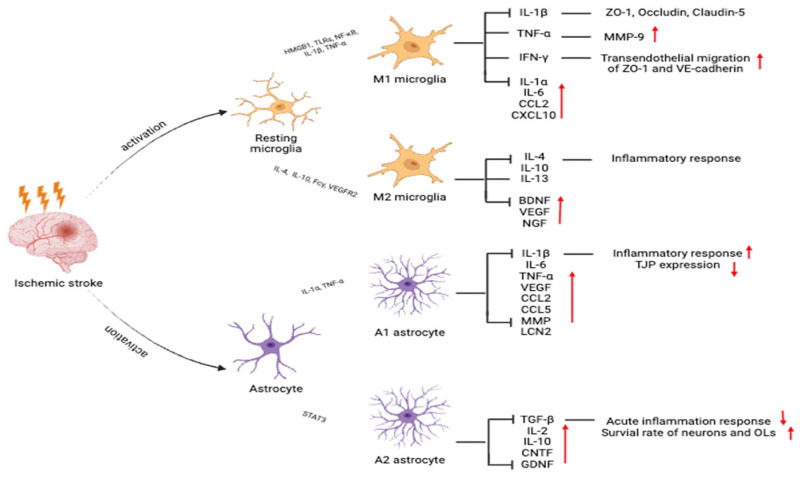
Activation states of microglia and astrocytes after ischemic stroke that affect neuroinflammation and repair. Microglia differentiate into the proinflammatory M1 or anti-inflammatory M2 phenotypes. Astrocytes are polarized into neurotoxic A1 or neuroprotective A2 subtypes. Red upward arrows (↑) indicate an increase in the corresponding cytokines, chemokines, or functional outcomes after ischemic stroke, while red downward arrows (↓) indicate a decrease.

Other cytokines, such as the interleukin-1 (IL-1) and interleukin-6 (IL-6) families, also exhibit pleiotropic effects during stroke. IL-1 β levels rapidly increase in the acute phase of ischemia, amplifying the inflammatory cascade and exacerbating neuronal damage (Figure 2). Conversely, interleukin-10 (IL-10), a key anti-inflammatory cytokine, exerts neuroprotection by suppressing inflammation, reducing apoptosis, and improving poststroke outcomes. These findings highlight the complex, context-dependent roles of cytokine signaling in ischemic pathophysiology and provide a foundation for the development of cytokine-targeted interventions in stroke therapy [131,132].

Chemokines are key players in orchestrating immune cell recruitment and amplifying neuroinflammatory responses following ischemic stroke. C-C motif chemokine ligand 2 (*CCL2*) and its receptor chemokine (C-C motif) receptor 2 (*CCR2*) are particularly well characterized. The CCL2/CCR2 axis facilitates the adhesion and transmigration of monocytes and other leukocytes across the BBB, thereby promoting their infiltration into the ischemic brain parenchyma. Elevated CCL2/CCR2 signaling is closely associated with increased infarct volume, enhanced neuroinflammation, and worsened neurological outcomes [133,134].

Furthermore, other chemokines, such as C-C motif chemokine ligand 3 (*CCL3*), C-C motif chemokine ligand 5 (*CCL5*), chemokine (C-X-C motif) ligand 1 (*CXCL1*), chemokine (C-X-C motif) ligand 2 (*CXCL2*), and chemokine (C-X-C motif) ligand 8 (*CXCL8*), contribute to stroke pathology by promoting neutrophil and Th1-polarized T cell infiltration. These chemokines are rapidly upregulated in response to ischemic injury and are critical in shaping the inflammatory milieu of the poststroke brain. Their concerted actions intensify tissue injury, disrupt the BBB, and perpetuate secondary damage [135].

Therefore, targeting chemokine signaling pathways is a promising therapeutic strategy for mitigating immune cell-mediated injury and limiting postischemic inflammation.

High mobility group box 1 (*HMGB1*), a key mediator of neuroinflammatory signaling, is markedly upregulated in the ischemic brain and plays a critical role in poststroke immune activation. Once released from necrotic or stressed neurons, HMGB1 binds to pattern recognition receptors, such as Toll-like receptors 2 and 4 (TLR2 and TLR4), leading to the activation of the NF-κB pathway and subsequently transcription of proinflammatory cytokines. This signaling cascade contributes to peripheral immune cell recruitment and neuroinflammation amplification [102,136].

TLR signaling also exhibits a dual role, depending on the timing and severity of the injury. Notably, TLR4 mediates the intensity of inflammatory responses in association with circulating lipopolysaccharide (LPS) levels, functioning as a critical regulator of immune homeostasis and damage propagation [136,137]. In parallel, MAPK pathways, including ERK, c-Jun N-terminal kinases (JNK), and p38, are rapidly activated in early-phase ischemia. These kinases promote the expression of inflammatory mediators and matrix metalloproteinases (MMPs), particularly MMP-9, which diminishes BBB integrity. The disruption of the BBB facilitates immune cell infiltration and intensifies cerebral edema and neuronal injury [138,139,140].

Targeting the HMGB1/TLR/NF-κB and MAPK/MMP signaling axes presents a promising therapeutic approach to alleviating neuroinflammation and preserving BBB function in ischemic stroke [141,142].

Microglia function critically in maintaining neural homeostasis following ischemic injury by clearing dead cells and cellular debris through phagocytosis. This process is primarily mediated by transmembrane protein 16F (TMEM16F) and triggering receptor expressed on myeloid cells 2 (TREM2) signaling pathways. While microglial phagocytosis is essential for limiting secondary damage and facilitating tissue repair, excessive or dysregulated phagocytic activity may facilitate synaptic pruning and neuronal loss, ultimately impairing cognitive function [143,144].

The complement system, particularly C1q and C3 activation, enhances microglial phagocytosis by tagging apoptotic cells and synapses for elimination. These complement components accumulate in the ischemic brain, where they interact with microglial receptors and promote phagocytosis [145,146]. The participation of both beneficial and detrimental phagocytic responses signifies that microglial activation and its regulation using TMEM16F, TREM2, and complement signaling constitute a complex and dynamic component of ischemic stroke pathology and recovery.

## 3. Structural and Functional Characteristics of MFG-E8

MFG-E8, also known as lactadherin, is a multifunctional glycoprotein that was first identified in milk fat globules and is expressed in various tissues [147], including the brain, liver, and kidneys, as well as in immune cells [26,30,148,149]. MFG-E8 contains an N-terminal EGF-like domain that includes an Arg-Gly-Asp (RGD) motif [149] responsible for binding integrins αvβ3 and αvβ5 [150,151]. Its C-terminal region consists of two discoidin domains (C1 and C2) [152,153,154,155], which mediate binding to phosphatidylserine (PS) exposed on the surface of apoptotic cells. Additionally, integrins, located on the outer membrane of phagocytes, act as receptors that recognize and bind to these discoidin domains, facilitating the engulfment of apoptotic cells [156,157,158,159].

This dual-binding capacity allows MFG-E8 to function as a bridging molecule between apoptotic cells and phagocytes, thereby facilitating the noninflammatory clearance of dying cells [150,155,160,161]. By interacting with integrins and PS, MFG-E8 activates downstream signaling pathways, including PI3K/Akt and ERK [161,162], which promote anti-inflammatory responses, cell survival, and tissue repair [152,163,164,165,166].

Unlike Annexin V, MFG-E8 binds to PS in a calcium-independent manner [167,168] and demonstrates high sensitivity, even at low PS concentrations [169,170,171,172]. Alternative splicing variants enriched in proline/threonine residues can modulate binding affinity and phagocytic activity [159,173], adding an extra layer of regulatory complexity [174,175]. These features position MFG-E8 as a crucial modulator in mitigating inflammation and maintaining tissue homeostasis [170,171,172,174,175].

### 3.1. Structural Features of MFG-E8: Domain Characteristics

MFG-E8 is a secreted glycoprotein first identified by Stubbs et al. [156] in 1990 as a novel cDNA sequence isolated from *Mus musculus* mammary epithelial cells. MFG-E8 was named for its high abundance in milk fat globules and its structural similarity to EGF and blood coagulation factors V and VIII [148,149]. MFG-E8 is a secreted protein 46–66 kDa in length that contains a signal peptide at the N-terminus [176], followed by two EGF-like domains (EGF1 and EGF2) and two C-terminal discoidin-like domains (C1 and C2) [152,153,154,155].

**Figure 3:**
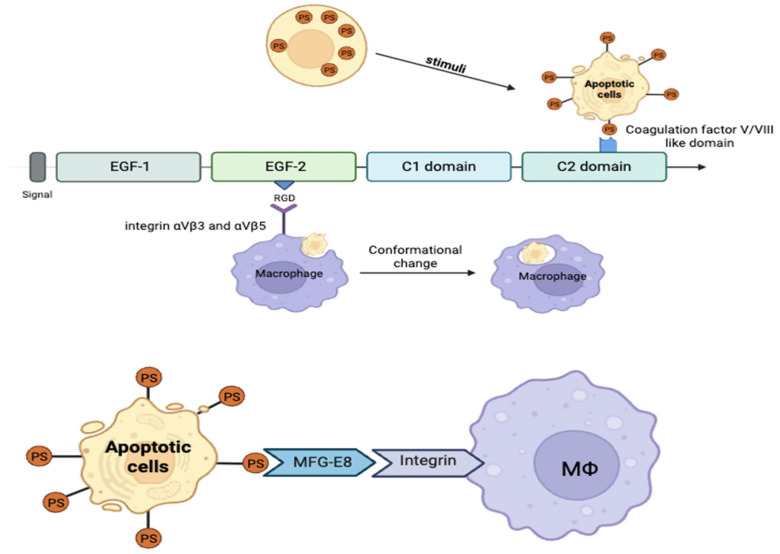
Structural domains of milk fat globule-EGF factor 8 (MFG-E8) and its functional mechanism. MFG-E8 binds phosphatidylserine on the surface of apoptotic cells using the C2 domain and integrins αvβ3/β5 using its RGD motif in the EGF-like domain to facilitate macrophage phagocytosis.

MFG-E8 typically contains a single EGF-like domain in humans, whereas two EGF-like domains are present in mice and bovines [31,176]. These EGF domains share homology with structural motifs found in Notch receptors and are implicated in cell growth and differentiation regulation [159,177]. The C1 and C2 discoidin-like domains are essential for binding to PS on the surface of apoptotic cells [152,153,155,159]. Specifically, the C1 domain mediates direct interaction with PS, whereas the C2 domain facilitates membrane association and facilitates cell adhesion and migration (Figure 3).

This domain configuration enables MFG-E8 to link apoptotic cells with phagocytes, thereby expediting the efficient clearance of cellular debris and contributing to anti-inflammatory and tissue repair processes [153,155,177].

### 3.2. Structural Characteristics of MFG-E8: Integrin-Binding Domain

The second EGF-like domain of MFG-E8 contains a conserved RGD motif, which specifically binds to αvβ3 and αvβ5 integrin receptors [149]. This interaction serves as a crucial molecular bridge that facilitates the recognition by macrophages of apoptotic cells, facilitating phagocytosis [156,157,158,159]. Through integrin binding, MFG-E8 activates multiple downstream signaling pathways, including AMPK/proto-oncogene tyrosine-protein kinase Src (Src) [178], Rac1 [165], and PI3K/Akt/mTOR [161], which collectively regulate phagocytosis, anti-inflammatory responses, angiogenesis, cell survival, and cell migration [163,164,165,177].

MFG-E8 exists in two splice variants. The longer isoform contains a proline/threonine-rich domain [159,173], which enhances the affinity of MFG-E8 for PS and potentiates phagocytic efficiency. In contrast, the shorter isoform lacks this domain and exhibits lower affinity for PS, resulting in reduced phagocytic activity [174,175]. This domain variation further modulates the functional capacity of MFG-E8 in immune regulation and tissue remodeling [168,179,180,181].

### 3.3. Structural Characteristics of MFG-E8: PS Binding Capacity and Therapeutic Potential

MFG-E8 exhibits a remarkably high affinity for PS [174,175], responding sensitively even to very low PS levels [169,170,171,172]. This high-affinity binding is distinct from that of Annexin V, which requires Ca^2+^ for PS interaction [168,182]. The calcium-independent PS binding by MFG-E8 not only differentiates it mechanistically from Annexin V but also indicates its broader role as a sensor of cellular stress beyond mere apoptotic cell recognition [165,183,184].

MFG-E8 binds preferentially to extracellular vesicles (EVs) characterized by highly curved membrane structures and PS exposure, indicating its involvement in EV function modulation [185]. These unique structural properties enable MFG-E8 to regulate several physiological processes, including cell adhesion, migration, immune modulation, and tissue homeostasis [35,161,165,178].

These multifaceted roles signify the considerable therapeutic potential of MFG-E8 as a target in diverse pathological conditions, from inflammatory diseases to tissue injury and repair. Its high sensitivity for PS detection and binding demonstrates its potential future clinical applications in modulating cell clearance, immune responses, and intercellular communication.

## 4. Functional Roles of MFG-E8 in the Central Nervous System

### 4.1. Phagocytic Function of MFG-E8 and Apoptotic Cell Clearance Mechanism

MFG-E8 is a critical bridging molecule that mediates the phagocytic clearance of apoptotic cells by recognizing PS as a lipid that is exposed on the surface of dying cells during apoptosis [150,155,160,161]. The F5/8C domain on the C-terminus of MFG-E8 binds specifically to PS [148], whereas the RGD motif in the N-terminal EGF-like domain interacts with αvβ3 and αvβ5 integrins expressed on phagocytes, thereby facilitating the efficient engulfment of apoptotic cells [149,150,151].

During apoptosis, exposed PS serves as a phagocytic “eat-me” signal to attract phagocytes, which are further guided by “find-me” signals released from dying cells [186]. This coordinated signaling ensures the rapid and precise clearance of cellular debris, thereby preventing secondary necrosis and subsequent inflammation [187]. Furthermore, chemokines such as chemokine (C-X3-C motif) ligand 1 (*CX3CL1*) upregulate MFG-E8 expression, thereby enhancing phagocytic activity [187]. MFG-E8 also cooperates with other receptors, such as T-cell membrane protein 4 (Tim-4), to orchestrate a two-step apoptotic cell removal process [188].

**Figure 4:**
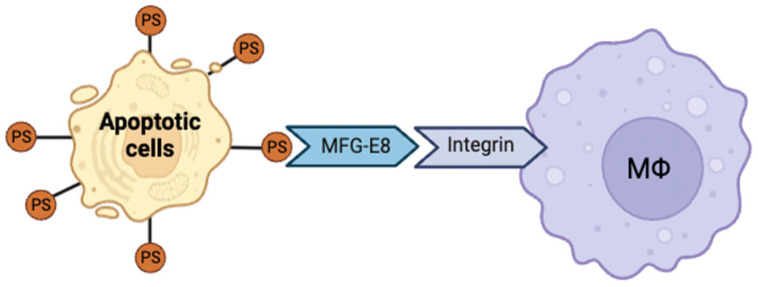
Mechanism of milk fat globule-EGF factor 8 (MFG-E8)–mediated apoptotic cell clearance. MFG-E8 bridges apoptotic cells and macrophages upon binding to phosphatidylserine (PS) and integrins, triggering signaling cascades that promote efferocytosis and inflammation resolution.

Conversely, structural alterations or domain deletions in MFG-E8 impair its PS binding and integrin-mediated phagocytic functions, leading to defective apoptotic cell clearance and exacerbated inflammatory response (Figure 4). Overall, MFG-E8 is an indispensable factor in maintaining tissue homeostasis and immune regulation through effective apoptotic cell clearance [152,189].

### 4.2. Anti-Inflammatory Mechanisms and Immunomodulatory Roles of MFG-E8

The efficient clearance of apoptotic cells is a noninflammatory process; however, delayed removal leads to secondary necrosis, causing the release of intracellular contents and damage-associated molecular patterns that activate immune cells and promote proinflammatory cytokine generation [150,155,160,161,187]. During this process, macrophages secrete inflammatory mediators, such as NO, prostaglandin E2, ROS, and cytokines (TNF-α, IL-1β, and IL-6), thereby amplifying inflammatory responses [190,191].

MFG-E8 attenuates inflammation by suppressing key signaling pathways, including NF-κB and MAPK, and modulates cytokine production using the suppressor of cytokine signaling-3 (SOCS3) [31] and STAT3 pathways [163]. Notably, MFG-E8 inhibits LPS-induced macrophage activation and competitively blocks integrin receptor binding of damage-associated molecules, such as HMGB1, thereby mitigating tissue injury [180,181].

MFG-E8 prevents excessive inflammatory activation and fosters an anti-inflammatory microenvironment conducive to tissue repair [179]. The anti-inflammatory effects of MFG-E8 have been demonstrated across various inflammatory disease models, establishing it as a critical immunoregulatory factor in alleviating chronic inflammation and tissue damage.

### 4.3. Regulation of Microglial Activation by MFG-E8

Microglia are the primary immune surveillance cells in the central nervous system that polarize into distinct phenotypes in response to inflammatory stimuli: the proinflammatory M1 phenotype or the anti-inflammatory, tissue-reparative M2 phenotype. MFG-E8 promotes microglial polarization toward the M2 phenotype, enhancing the secretion of anti-inflammatory cytokines and activating phagocytosis of damaged neural tissue, thereby fostering a neuroprotective and neuroregenerative environment [192,193].

While M1 microglia secrete proinflammatory cytokines that exacerbate neuronal injury, MFG-E8-mediated M2 activation counteracts this effect and helps restore neuroinflammatory balance. Furthermore, MFG-E8 restores the phagocytic capacity of microglia, facilitating the clearance of apoptotic debris and suppressing proinflammatory signaling pathways, thereby mitigating chronic inflammation [193,194].

Thus, MFG-E8 functions as a critical regulator of microglial functional states by modulating neuroinflammation and promoting neural repair processes.

### 4.4. Regulation of Astrocyte Reactivity by MFG-E8

Astrocytes not only provide structural support within the nervous system but are also key players in the regulation of neuroinflammatory responses. Upon inflammatory stimuli, astrocytes polarize into two major phenotypes: neurotoxic A1 and neuroprotective A2. A1 astrocytes intensify neuronal injury through the secretion of proinflammatory cytokines, whereas A2 astrocytes promote tissue repair and neuroprotection by releasing anti-inflammatory cytokines and neurotrophic factors [195,196,197].

MFG-E8 promotes the activation of the A2 astrocytic phenotype, thereby fostering an anti-inflammatory environment and facilitating the recovery of damaged neural tissue. Furthermore, MFG-E8 interacts with microglia to regulate neuroinflammation and neuroregenerative processes, highlighting its integrative role in central nervous system homeostasis [187,198].

These functions indicate the potential of MFG-E8 as a therapeutic target for modulating astrocyte-mediated neuroinflammatory responses and enhancing neural repair mechanisms following central nervous system injury and disease.

### 4.5. Regulatory Mechanisms of MFG-E8 Expression

The expression of MFG-E8 is tightly regulated by multiple transcriptional and post-transcriptional mechanisms. The chemokine *CX3CL1* has been identified as a key upstream regulator that induces MFG-E8 transcription in microglia, with its activity closely linked to inflammatory transcription factors such as NF-κB and STAT3 [163,187]. Beyond transcriptional regulation, recent studies have demonstrated that nanoparticle-mediated microRNA (miRNA) delivery can modulate MFG-E8 expression in macrophages. For example, gold-core nanoparticles coated with *miR-99b* suppressed MFG-E8 protein levels in gut macrophages, thereby influencing enterocyte migration and local immune responses. In addition, macrophage-like nanoparticles have been developed to absorb endotoxins and proinflammatory cytokines, providing mechanistic insights into the functional networks of MFG-E8 and its role in macrophage-mediated inflammatory regulation. These findings underscore the importance of post-transcriptional regulation and tissue-specific contexts in shaping MFG-E8 function. Future studies should expand on these insights by applying transcriptomic profiling in ischemic stroke models, with particular attention to promoter regulation, splicing variants, and the interplay between transcription factors and miRNA [199,200].

At the protein level, MFG-E8 executes its biological functions by binding PS exposed on apoptotic cells while simultaneously engaging αvβ3 and αvβ5 integrins on phagocytes [150,155,160,161]. This bridging interaction activates downstream signaling cascades, including ERK1/2, p38 MAPK, and JNK, which collectively suppress proinflammatory cytokine production and enhance anti-inflammatory responses, thereby establishing a homeostatic feedback loop [201]. Conversely, reduced expression or disruption of MFG-E8 signaling impairs apoptotic cell clearance, leading to the accumulation of apoptotic debris, chronic inflammation, and subsequent tissue damage. Thus, delineating the precise molecular regulation of MFG-E8 expression and signaling remains critical for the development of targeted therapeutic strategies for inflammatory and neurodegenerative diseases.

In parallel, dysregulated MFG-E8 expression has been observed in tumor biology. Acting as a molecular bridge between PS and integrins, MFG-E8 is frequently overexpressed in a range of cancers and has been implicated in diverse processes, including attenuation of inflammation, induction of regulatory T cells (Tregs), promotion of efferocytosis, angiogenesis, allograft tolerance, and metastatic progression. Notably, MFG-E8 overexpression correlates with poor prognosis in breast, colorectal, and esophageal cancers [202,203,204]. Yamada et al. [205] further demonstrated that MFG-E8 promotes angiogenesis by upregulating VEGF and endothelin-1 (ET-1) in bone marrow-derived mesenchymal stromal cells, thereby facilitating melanoma progression. Moreover, MFG-E8 fosters M2 polarization of macrophages, while its blockade enhances antitumor effector T-cell responses and suppresses Tregs, resulting in tumor regression.

Taken together, these findings indicate that MFG-E8 exerts dual roles in cancer development and progression, with its effects shaped by cancer type, tumor microenvironment, and interactions between malignant and immune cells. However, its systemic impact on antitumor immunity remains incompletely understood, underscoring the need for more comprehensive mechanistic studies and rigorous safety evaluations to inform the clinical translation of MFG-E8-based interventions.

### 4.6. MFG-E8-Related Biomarkers: Potential Indicators and Clinical Applicability

Research on MFG-E8-related biomarkers remains in its early stages, and clinical validation is currently limited. Despite this, recent studies have identified several promising candidates that may serve as indicators of disease progression and therapeutic response. Concentrations of MFG-E8 in plasma and cerebrospinal fluid (CSF) have been shown to vary depending on the pathological context. For example, CSF MFG-E8 levels are decreased in patients with cerebral amyloid angiopathy (CAA) or Alzheimer’s disease, whereas plasma levels may be elevated in certain cancers. These context-dependent variations suggest that plasma and CSF MFG-E8 levels could provide valuable information for prognosis and the development of targeted therapeutic strategies [206,207].

MFG-E8 also interacts with PS-exposing cells and EVs, promoting efferocytosis and modulating immune responses. EV-associated MFG-E8 may serve as a surrogate marker for macrophage and microglial activation, reflecting therapeutic efficacy in neurological disorders [208]. In addition, MFG-E8 has been shown to influence microglial polarization, shifting cells from the proinflammatory M1 phenotype to the anti-inflammatory M2 phenotype. This shift is accompanied by reductions in M1-associated cytokines such as TNF-α, IL-1β, and IL-6, along with increases in M2 markers including Arg-1, IL-10, and CD206. Such modulation of inflammatory responses underscores the therapeutic potential of MFG-E8 in a variety of neuroinflammatory conditions [209].

To establish the clinical utility of these biomarkers, future preclinical and clinical studies are needed to systematically assess plasma and CSF MFG-E8 levels, PS-exposing EVs, and microglial activation markers. These investigations will be critical for patient stratification and for monitoring the effectiveness of MFG-E8-based therapeutic interventions.

## 5. MFG-E8 Expression in Ischemic Stroke Models

Ischemic stroke triggers a complex cascade of events, including cell death [210], inflammation [211], energy depletion [212], ionic imbalance [213], excitotoxicity [214], and oxidative stress [215], which collectively initiate inflammatory and immune responses [216]. These processes ultimately result in irreversible brain damage. Following successful reperfusion, blood flow restoration to the ischemic brain can paradoxically induce secondary reperfusion injury [217]. Reperfusion promotes ROS generation, amplifies inflammatory and immune responses, and induces excessive neuronal death, BBB disruption, and innate and adaptive immune system activation, thereby exacerbating brain injury [218]. These complex pathological processes contribute to the progression of ischemic brain injury. To counteract these detrimental mechanisms, various biomolecules and signaling pathways that promote neuroprotection and inflammation regulation have been extensively investigated [210]. MFG-E8, which is expressed by astrocytes and microglia in the brain, is crucial in attenuating secondary injury after cell death by mitigating inflammatory responses and tissue damage [219]. Notably, MFG-E8 binds to PS exposed on the surface of apoptotic cells and activates macrophage αvβ3/5 integrin receptors, thereby facilitating efficient efferocytosis [161,166,187]. Moreover, MFG-E8 is an important neuroinflammation and neuronal apoptosis modulator following ischemic injury [220,221]. MFG-E8 suppresses inflammatory responses and exerts tissue-protective effects, not only in ischemic stroke but also in intestinal ischemia [198] and Alzheimer’s disease models [139]. These findings demonstrate the significant neuroprotective potential of MFG-E8 in ischemic stroke, highlighting its promise as a novel therapeutic target for stroke treatment (Table 1).

Preclinical ischemic stroke models indicate that MFG-E8, primarily expressed by astrocytes and microglia, is upregulated in peri-infarct regions, coinciding with apoptotic cell accumulation and microglial activation. MFG-E8 binds PS on apoptotic cells and αvβ3/αvβ5 integrins on phagocytes, facilitating selective efferocytosis while suppressing proinflammatory signaling [221]. Comparative analyses with other PS-binding proteins reveal distinct advantages: Annexin V and PS-targeting antibodies inhibit phagocytosis but may exacerbate inflammation and delay tissue repair; Gas6 and Protein S activate TAM receptors to promote clearance and anti-inflammatory signaling but risk pathological angiogenesis and immunosuppression. In contrast, MFG-E8 coordinates apoptotic cell removal with anti-inflammatory responses, enhances neuronal survival, supports vascular remodeling, and demonstrates favorable stability and CNS targeting via exosome-based or intranasal delivery. These properties position MFG-E8 as a promising therapeutic candidate for ischemic stroke, though further investigation into potential adverse effects and drug resistance is warranted [221,222].

**Table 1:** Functional roles of MFG-E8 after ischemic stroke in animal models.

Function	Potential Mechanism	Reference
Inflammation modulation	Suppresses cytokine release and promotes M2 macrophage polarization	[35]
Anti-apoptosis	Regulates Bax/Bcl-2 expression and caspase-3 inhibition	[27]
Efferocytosis promotion	Enhances efferocytosis by αvβ3 integrin binding	[186,213]
Neuroprotection	Promotes tissue repair and reduces infarct volume	[27,113]
Neurogenesis	Stimulates neural stem cell proliferation and migration	[223]

Administration of rhMFG-E8 at 160 μg/kg via intravenous injection into the tail vein significantly reduced infarct volume in the ischemic penumbra to 25% at 24 h post-surgery in adult male Sprague–Dawley rats subjected to permanent middle cerebral artery occlusion (MCAO), a well-established rodent model of focal cerebral ischemia [27]. rhMFG-E8 markedly suppressed apoptosis markers, including cleaved caspase-3, Bcl-2, and Bax, and attenuated proinflammatory cytokines such as IL-6 (decreased by 39.6%) and TNF-α, while increasing the Bcl-2/Bax ratio by 51.9% compared to vehicle-treated animals [27]. Functionally, rhMFG-E8 improved neurological outcomes, reflected by significantly lower modified Neurological Severity Scores (mNSS) at days 4, 8, 11, and 15 post-stroke. Immunofluorescence analysis revealed increased RECA-1-positive endothelial cells in the peri-infarct region and higher numbers of BrdU- and DCX-positive cells in the subventricular zone (SVZ), indicating both neuroprotective and neuroregenerative effects [25]. These findings were obtained from controlled, randomized preclinical studies in rats, providing mechanistic evidence for rhMFG-E8-mediated suppression of apoptosis and inflammation following ischemic stroke.

These findings demonstrate the significant potential of MFG-E8 as a novel therapeutic target for ischemic stroke. Therapies that harness the neuroprotective and anti-inflammatory effects of MFG-E8 may offer an innovative approach to stroke treatment.

However, most studies involved small sample sizes and lacked blinding and randomization, limiting the interpretability of results. Additionally, species-specific differences in MFG-E8 domain structure warrant caution in extrapolating animal data to humans. Future research should provide quantitative comparisons across diverse models, dosages, and time points.

## 6. MFG-E8 in Ischemic Stroke Therapy: Limitations

Tag-free rhMFG-E8 can be lyophilized for long-term storage while retaining full biological activity upon reconstitution. Preclinical studies indicate favorable pharmacokinetics and safety: administration of up to 2 mg/kg in mice over 28 days showed no toxicity, inflammation, or body weight changes, and endotoxin levels were significantly below industry standards. Nevertheless, several limitations remain that should be considered in interpreting current findings and designing future studies [224].

Most preclinical investigations involve small sample sizes and often lack comprehensive pharmacokinetic, pharmacodynamic, and tissue distribution data. Assessments of BBB permeability and systemic toxicology are limited, leaving unresolved concerns regarding potential adverse effects, including immune dysregulation, tumor promotion via angiogenesis, and interference with normal apoptotic cell clearance [225,226]. Differences in MFG-E8 domain structure across species, lack of blinding and randomization, and variability in experimental models further constrain the generalizability of findings. Moreover, clinical data remain scarce, limiting translational potential.

To date, validated quantitative ranges of MFG-E8 in plasma or CSF of human ischemic stroke patients have not been established. Consequently, no standardized reference ranges are currently available for use in stroke cohorts. However, MFG-E8 levels reported in other neurological disorders may provide contextual insight. For instance, CSF MFG-E8 levels in patients with CAA were significantly lower than those in healthy controls (*p* = 0.01) and AD patients (*p* < 0.001) [227], suggesting that MFG-E8 concentrations may vary according to the type of neurological disorder. In summary, while MFG-E8 levels appear to differ across neurological conditions, validated quantitative ranges in ischemic stroke patients have not yet been reported.

Sex-specific differences in stroke pathophysiology and therapeutic responses represent another critical consideration. Women often present with atypical symptoms, experience delayed hospital arrival, and may have a higher risk of severe stroke compared to men [228]. Current data on MFG-E8 expression, signaling, and therapeutic efficacy across sexes are limited. Future studies should comprehensively evaluate immune responses, neuroprotection, and inflammatory biomarkers to inform sex-specific therapeutic strategies.

Finally, the context-dependent risks of MFG-E8-mediated angiogenesis should be addressed. The integrin-binding motif within MFG-E8′s EGF-like domain promotes cell adhesion, angiogenesis, and vascular remodeling [150,155,160,161]. While supporting tissue repair and apoptotic cell clearance, overexpression has been associated with tumor progression and an immunosuppressive microenvironment in certain cancers, whereas inhibition enhances antitumor T cell activity [229].

However, its systemic impact on antitumor immunity remains incompletely understood, underscoring the need for more comprehensive mechanistic studies and rigorous safety evaluations to inform the clinical translation of MFG-E8-based interventions.

These properties may theoretically exacerbate hemorrhagic transformation in stroke or tumor progression in individuals with latent neoplasia, emphasizing the need for careful evaluation of safety alongside therapeutic efficacy [202,204].

## 7. MFG-E8 in Ischemic Stroke Therapy: Prospects for Improvement

Advanced delivery strategies are under active investigation to overcome the aforementioned limitations. MFG-E8 can cross the BBB via exosome-mediated transport, supporting intranasal or intravenous exosome delivery as a promising strategy for sustained therapy. Small peptide derivatives, such as MSP68, may also penetrate the BBB, although their effects on neural stem cells (NSCs) require further evaluation. Engineering EVs with fusion proteins, such as the PS-binding domain of MFG-E8 linked to an RGD-4C peptide, has been shown to enhance targeted delivery to the ischemic brain by approximately 2.5-fold compared to naïve EVs [230,231].

Hypoxia-preconditioned human embryonic stem cell-derived EVs (hESC-HypoxEVs) exhibit enhanced glutathione redox capacity and potentiate senotherapeutic effects on NSCs. Administration of hESC-HypoxEVs precoated with MFG-E8 significantly increased NSC and newborn neuron populations in the subventricular zone and improved sensorimotor recovery in a rat ischemic stroke model [166,185].

Collectively, these findings highlight the potential of MFG-E8-based delivery systems to improve stability, targeting, and functional efficacy. Future research should provide quantitative comparisons across diverse models, dosages, and time points to enable robust evaluation of therapeutic outcomes. To establish the clinical utility of these biomarkers, future preclinical and clinical studies are needed to systematically assess plasma and CSF MFG-E8 levels, PS-exposing EVs, and microglial activation markers. These investigations will be critical for patient stratification and for monitoring the effectiveness of MFG-E8-based therapeutic interventions.

Future studies should also focus on optimizing dosing, administration routes, and combination strategies while rigorously addressing safety, pharmacokinetics, and long-term efficacy, ultimately facilitating clinical translation of MFG-E8-related therapeutics.

## 8. Discussion

With the rapid progression of global population aging, the incidence and socioeconomic burden of ischemic stroke are expected to rise substantially [232]. Aging is associated with vascular endothelial dysfunction, chronic low-grade inflammation, and increased vulnerability to cerebrovascular injury, complicating stroke pathophysiology and limiting the efficacy of current reperfusion-based therapies [233,234]. Against this backdrop, MFG-E8 has emerged as a promising therapeutic candidate due to its dual roles in apoptotic cell clearance and inflammation resolution. Positioned at the interface of immune regulation and tissue remodeling, MFG-E8 has demonstrated consistent preclinical efficacy in reducing infarct volume, attenuating neuroinflammation, and improving neurological and functional outcomes.

MFG-E8 shows potential not only as a monotherapy but also in combination with thrombolytic, neurorestorative, or advanced cell-based therapies. Its ability to promote long-term neuroregeneration further underscores the need for evaluation in chronic stroke models, where functional recovery remains a major clinical challenge.

To facilitate clinical translation, a structured and stepwise research roadmap is essential. Key priorities include the following: evaluating safety, pharmacokinetics, long-term functional recovery, and neuroregenerative effects in large-animal models; conducting preclinical and early-phase clinical trials to assess both monotherapy and combination strategies; validating translational biomarkers, such as plasma or cerebrospinal fluid MFG-E8 levels, phosphatidylserine-exposing extracellular vesicles, and microglial activation markers, for patient stratification and monitoring therapeutic responses; and developing stable formulations with efficient central nervous system-targeted delivery systems. Through these integrated approaches, MFG-E8-based therapeutics have the potential to advance from preclinical models to clinical application, offering a next-generation strategy for the treatment of ischemic stroke.

## 9.Methods

This review synthesizes the current knowledge on the functions of MFG-E8 in ischemic stroke and its regulatory mechanisms in neuroinflammation. A systematic search of the literature published from 1990 to 2025 was conducted on the PubMed, Scopus, and Web of Science databases. Keywords such as “ischemic stroke”, “MFG-E8”, “neuroinflammation”, “immune response” and “apoptotic cells” were used for the search and combined using Boolean operators (AND, OR, and NOT). The inclusion criteria were peer-reviewed English studies that investigated the relationship between MFG-E8 and ischemic stroke or elucidated the experimental/clinical mechanisms of inflammation regulation. Selected studies were thematically analyzed to evaluate the neuroprotective and anti-inflammatory roles of MFG-E8 in ischemic stroke, identify knowledge gaps, and determine future research directions.

## 10. Conclusions and Future Directions

The rising prevalence of ischemic stroke and limitations of current therapies highlight the need for novel interventions. MFG-E8, with its multifaceted roles in modulating inflammation, enhancing efferocytosis, and promoting tissue repair, emerges as a promising therapeutic candidate. Key actionable priorities include the following:

(1) Standardize preclinical protocols: Optimize dosing, administration routes, and pharmacokinetic/pharmacodynamic (PK/PD) readouts in large animal stroke models.
(2) Validate biomarkers: Quantify blood/CSF MFG-E8 and EV-associated markers as reliable pharmacodynamic and response indicators.
(3) Define safety margins: Assess risks of hemorrhagic transformation and potential tumor-related effects with prospectively specified monitoring.
(4) Conduct sex-stratified analyses: Evaluate efficacy and safety separately in male and female preclinical models.
(5) Establish first-in-human criteria: Determine optimal timing relative to reperfusion therapy and compatibility with MT/rt-PA.

These priorities provide a structured framework to guide the translation of MFG-E8-based therapies from preclinical studies to clinical application and ultimately reduce the neurological and societal burden of ischemic stroke.

## Data Availability

Details are available from the authors.

## Abbreviations

Akt	Protein Kinase B
AMPA	α-amino-3-hydroxy-5-methyl-4-isoxazolepropionic acid
AMPK	AMP-activated protein kinase
Apaf-1	Apoptotic protease activating factor 1
ARE	antioxidant response element
ASK1	apoptosis signal-regulating kinase 1
ATP	Adenosine triphosphate
BAX	Bcl-2-associated X protein
BBB	blood–brain barrier
Bcl-2	B-cell lymphoma 2
BDNF	brain-derived neurotrophic factor
BrdU	5-Bromo-2′-deoxyuridine
C1, C2	two C-terminal discoidin-like domains
Ca^2+^	calcium ions
CAA	cerebral amyloid angiopathy
Casp8	caspase-8
Casp9	caspase-9
CCL2	C-C motif chemokine ligand 2
CCL3	C-C motif chemokine ligand 3
CCL5	C-C motif chemokine ligand 5
CCR2	chemokine (C-C motif) receptor 2
CXCL1	chemokine (C-X-C motif) ligand 1
CXCL2	chemokine (C-X-C motif) ligand 2
CXCL8	chemokine (C-X-C motif) ligand 8
cIAPs	cellular inhibitor of apoptosis proteins
CK2	Casein kinase 2
CREB	cAMP response element-binding protein
CSF	cerebrospinal fluid
CX3CL1	chemokines such as chemokine (C-X3-C motif) ligand 1
CYT C	cytochrome c
DAPK1	death-associated protein kinase 1
DCX	Doublecortin
EGF1, EGF2	EGF-like domains
ERK	Extracellular signal-regulated kinases
ET-1	endothelin-1
EVs	extracellular vesicles
FADD	Fas-associated protein with a death domain
GPX4	impaired glutathione peroxidase 4
GSK3β	glycogen synthase kinase 3
hESC-HypoxEVs	human embryonic stem cell-derived EVs
HIF-1α	hypoxia-inducible factor-1 alpha
HMGB1	High mobility group box 1
I/R	Ischemia–reperfusion
IL-1	interleukin-1
IL-1 β	interleukin-1β
IL-10	interleukin-10
IL-6	interleukin-6
IRS-1	Insulin receptor substrate-1
IS	Ischemic stroke
JNK	c-Jun N-terminal kinases
K	potassium
Keap1	Kelch-like ECH-associated protein 1
LPS	lipopolysaccharide
MAPK	mitogen-activated protein kinase
MCAO	middle cerebral artery occlusion
MEM16F	transmembrane protein 16F
MFG-E8	Milk fat globule-EGF factor 8
miRNA	microRNA
MMPs	matrix metalloproteinases
MT	mechanical thrombectomy
mTOR	Mechanistic target of rapamycin
mTORC1	mechanistic/mammalian target of rapamycin complex 1
Na^+^/K^+^-ATPase	sodium–potassium adenosine triphosphatase
NF-κB	Nuclear factor kappa B
NMDARs	N-methyl-D-aspartate receptors
nNOS	neuronal nitric oxide synthase
NO	nitric oxide
Nrf2	nuclear factor erythroid 2-related factor 2
NSCs	neural stem cells
PDK1	3-Phosphoinositide-dependent protein kinase-1
PI3K	Phosphoinositide 3-Kinase
PPAR-γ	peroxisome proliferator-activated receptor gamma
pro-Casp8	procaspase-8
pro-Casp9	procaspase-9
PS	phosphatidylserine
PSD95	Postsynaptic density protein 95
PTEN	Phosphatase and tensin homolog
r-tPA	recombinant tissue plasminogen activator
Rac1	Rac Family Small GTPase 1
RGD	Arg-Gly-Asp
rhMFG-E8	recombinant human MFG-E8
RIP1	receptor interacting protein 1
RIP3	receptor interacting protein 3
ROS	reactive oxygen species
SOCS3	suppressor of cytokine signaling-3
solTNF	soluble TNF
Src	proto-oncogene tyrosine-protein kinase Src
STAT3	Signal transducer and activator of transcription 3
SVZ	subventricular zone
tBID	truncated BID
Tim-4	T-cell membrane protein 4
TLR2, TLR4	Toll-like receptors 2 and 4
tmTNF	transmembrane TNF
TNF-R1	tumor necrosis factor receptor 1
TNFR1	through TNF receptor 1
TNFR2	TNF receptor 2
TNFα	tumor necrosis factor alpha
TRADD	TNF receptor-associated death domain
TRAF2/5	TNF receptor-associated factors 2 and 5
Tregs	regulatory T cells
TREM2	triggering receptor expressed on myeloid cells 2
